# Functions of the right DLPFC and right TPJ in proposers and responders in the ultimatum game

**DOI:** 10.1093/scan/nsz005

**Published:** 2019-01-28

**Authors:** Constantin Speitel, Eva Traut-Mattausch, Eva Jonas

**Affiliations:** Department of Psychology, University of Salzburg, Hellbrunner Strasse, Salzburg, Austria

**Keywords:** tDCS, self-regulation, perspective taking, self-other-distinction

## Abstract

Recent studies explored a network of brain regions involved in economic decision making. The present study focuses on two of those regions, each relevant for specific and distinct functions in economic decision making: the right temporoparietal junction (rTPJ) and the right dorsolateral prefrontal cortex (rDLPFC). In two experiments using transcranial direct current stimulation, we explored two proposed functions of these areas in bargaining situations using the ultimatum game (UG): understanding the others perspective and integration of fairness norms. Participants first took the role of the proposer and then the role of the responder. We showed that stimulation of the rTPJ only affected the proposer condition. Interestingly, inhibition of the rTPJ led to fairer offers, which strengthens the view that the role of the rTPJ in bargaining situations is to differentiate one’s own from the other’s perspective. Furthermore, we argue that the rDLPFC is most likely involved in suppressing self-interest when a person is confronted with a direct reward but does not play a role in long-term reward anticipation or integrating social fairness norms. We conclude that self-interest inhibition is shown only in responders, and that perspective taking seems to be a necessary specifically for proposers in the UG.

## Introduction

To understand human decision making during bargaining, the ultimatum game (UG) is an often used tool (Guth *et al.*, 1982). In the UG, one player (the proposer) receives an amount of money that she or he has to divide between herself or himself and another player (the responder). The proposer decides how the whole sum of money will be split between the two players. Afterwards, the responder has the option to either accept or reject the offer. If the responder accepts, the split is made according to the proposer’s offer. However, if the responder rejects, both parties receive nothing (Fehr and Camerer, 2007; Sanfey, 2007). According to the idea of utility maximization, the most economically rational solution for proposers would be to give the smallest amount of money possible to the responder, and the most rational solution for the responder would be to accept any offer, because any profit is better than none (Camerer and Thaler, 1995). Yet, research has identified a contradictory pattern of behaviour; most proposers offer ~50% of the total amount of money and offers of 20% or less of the total amount are usually rejected by responders (Sanfey *et al.*, 2003; Fehr and Camerer, 2007; Kirman and Teschl, 2010). This is especially true when participants interact (or believe they are interacting) with real agents but less so in the case of a computer opponent (van’t Wout *et al.*, 2006). It is suggested that both proposers and responders consider further information, such as the fairness of the offer itself, and that they are aware of those fairness norms within the respectable others mind (Guroglu *et al.*, 2011).

There is a growing literature about these neuronal underpinnings of these social cognitions (Sellaro *et al.*, 2016). To make causal statements about the involvement of brain regions, we used transcranial direct current stimulation (tDCS) to manipulate neuronal activity. This is an easy-to-apply method in which an electric current passes through two electrodes (one cathode and one anode) that are placed on the scalp. Results of previous studies suggest that anodal stimulation leads to a stronger activation and cathodal stimulation to an inhibition of the stimulated brain area (Been *et al.*, 2007; Nitsche *et al.*, 2008). The approach also allows for a very good control condition without any stimulation, the sham condition, which induces the same feeling of being stimulated without an actual direct current. In the present study, we focused on the following two brain areas that are important for functions necessary for bargaining: the temporoparietal junction (TPJ) and the dorsolateral prefrontal cortex (DLPFC). We have chosen these regions because they are part of the network involved in interaction in economic decision making (Stallen and Sanfey, 2013) and at the same time, very well accessible to the tDCS stimulation (Thair *et al.*, 2017). Our goal was to show that these areas have distinctive functions in economic decision making, namely, perspective taking and emotion regulation, which we will explain in the next two paragraphs, respectively. In two experiments, we set out to clarify the role of the right TPJ (rTPJ; Experiment 1) and the right DLPFC (rDLPFC; Experiment 2) by using the UG.

Several studies reported that the TPJ is often activated during perspective taking; understanding others’ goals, intentions and desires; reasoning about the actions of others; and appreciating differences between one’s own and others’ perspectives as well as during conflicts between those perspectives (Perner *et al.*, 2006; Singer, 2006; Decety and Lamm, 2007; Schulte-Ruther *et al.*, 2008; Van Overwalle, 2009; Hetu *et al.*, 2012; Morelli and Lieberman, 2013). Studies showed that the understanding of another person’s thoughts and beliefs is required in strategic financial interactions (Kirman and Teschl, 2010; Artinger *et al.*, 2014). Artinger *et al.* (2014) for example, demonstrated that it was not emotional empathy but cognitive perspective taking that was highly correlated with offers proposed by dictators in the dictator game. They concluded that perspective taking can be used to pursue both self-interest and pro-social goals. Recent studies showed that the rTPJ is actively involved in economic decision making when participants have information about the other player (Rilling *et al.*, 2004; Baumgartner *et al.*, 2014). Morishima *et al.* (2012) showed with functional magnetic resonance imaging that the grey matter volume of specifically the rTPJ correlates highly with actions favouring equality. In a reciprocity task, they demonstrated that participants did not become purely altruistic but that the rTPJ was recruited when the conflict between their own material payoff and the principle of equality was greatest. Others demonstrated that impairment of the rTPJ leads to an impaired ability to distinguish between information relevant to oneself and information relevant to another person (Decety and Sommerville, 2003). If this ability is impaired, participants may resort to a simple heuristic of sharing equally. Supporting this explanation are results from children around the age of 5 years playing the UG. These younger children, with established social heuristics or norms but an underdeveloped perspective-taking ability, often made more offers close to the fair solution (i.e. equal split) compared to older children with well-developed perspective-taking skills (Leventhal *et al.*, 1973; Kanngiesser and Warneken, 2012). Even though these results point to the relevance of the rTPJ, to our knowledge, no study has assessed the causal link to the rTPJ in the UG.

We hypothesized that proposers whose rTPJ is inhibited via tDCS will be more biased in the direction of an equal split compared to participants in a sham condition in the UG. We reasoned that if the ability to distinguish their own interests from the other player’s is disturbed, participants will fall back on simple rules of equality to solve this conflict, like when their perspective-taking abilities were not fully developed. For responders we assumed, with the same reasoning, that if participants consider the proposer’s perspective during their rejection decision, they should reject more offers that deviate from an equal split. Parallel to recent studies we also included an anodal stimulation for the responder condition (Blair-West *et al.*, 2018) as well as for the proposer condition. Our hypothesis was that an increase in rTPJ activity should increase the participant’s ability for self-other distinction and thus reduce the number of proposed and accepted equal split solutions in the UG.

The second region we focused on, the rDLPFC, is strongly associated with self-control (Hare *et al.*, 2009), response selection (Hadland *et al.*, 2001) and other executive functions (Duncan and Owen, 2000). Knoch *et al.* (2006) used neuromodulation techniques such as repetitive transcranial magnet stimulation or tDCS (Knoch *et al.*, 2008) to decrease the activity of the rDLPFC and observed its effect on the UG. Participants played multiple UGs against multiple anonymous others. They found that an inhibition of rDLPFC activity in responders was related to an increased acceptance of unfair offers in the UG (Knoch *et al.*, 2006). It was demonstrated that this effect was specific for human interaction. Others, like Civai *et al.* (2015) found the same result is true for the neighbouring medial prefrontal cortex, but only if the stimulated person makes the decision for themselves, not if they decide for a third party. Therefore, it seems unlikely that these frontal areas are involved in perspective taking for bargaining situations. These studies provide two possible explanations of this behaviour: first, that the rDLPFC may be responsible for the top-down control that modulates the self-interested impulses and integrates participants’ fairness norms into their behaviour (Knoch *et al.*, 2006), and second, that the rDLPFC may control negative emotional reactions to the unfair offers, since it is related to emotion regulation (Grecucci *et al.*, 2013), a part of the self-control function of the DLPFC. Morewedge *et al.* (2014) argued, for example, that rejections constitute a failure to inhibit a desire to punish the first player for making an unfair offer. They found that intoxicated participants were more likely to reject unfair offers than sober participants. As intoxication tends to exacerbate a decision maker’s prepotent response, this result provides more support for the self-control than the altruistic punishment account. Other research from social cognitive neuroscience supports this finding (Tabibnia *et al.*, 2008).

Altogether, for the present study, we expect responders to accept more unfair offers when cathodal stimulation is applied to the rDLPFC, parallel to previously reported findings (Knoch *et al.*, 2008). In addition, we would argue that if the integration of fairness norms is generally impaired by rDLPFC disruption, proposers should also be affected. More specifically, when making an offer to another player, participants also need to weight their own self-interest against their norms of equality. We also include an anodal stimulation condition for the stimulation of the rDLPFC. Increasing the activity of the rDLPFC should lead to a better ability to integrate fairness norms and therefore a lower acceptance rate. If impaired emotion regulation as a reaction to unfair treatment by the other player is the cause, proposers should not be affected by either stimulation condition, since the participants would not be reacting to a negative or aversive stimulus. . In summary, we assumed that responders who receive cathodal stimulation over the rDLPFC will accept more unfair offers than sham stimulation participants, in keeping with the results of Knoch *et al.* (2008). Our hypothesis for proposers is that if the cathodal stimulation of the rDLPFC impacts fairness-norm integration, offer size should decrease because self-interest should have a stronger impact on the behaviour.

Finally, the objective of combining the two experiments in one study was to highlight the differences between the functions of the targeted brain areas. Since we predict the same outcome for the responder condition, one cannot dissociate between the brain areas. However, in the proposer condition, a directly opposite effect is to be expected. If the distinction between the own and the others outcome is regulated by the rTPJ, inhibition would lead to more equal offers in the proposer condition. If the rDLPFC has the suggested function of integrating fairness norms, inhibiting the rDLPFC of proposers should lead to more unfair offers.

## Experiment 1: rTPJ

### Methods

#### Participants

Seventy-eight healthy participants (40 females), mean age 23.74 years (s.d. = 6.36), gave written informed consent and were instructed about the general procedure and potential side effects of tDCS. The study was approved by the ethics committee of the University of Salzburg. Additionally, it was explained that the participants’ data would be treated anonymously and that participants could stop their participation in the experiment whenever they wanted without giving an explanation. As a reward, participants received the money earned in the UG. Eleven participants had to be excluded from the analysis: three because they did not believe the cover story and thought that they played against a computer, and eight had to be excluded because of technical problems.

#### Procedure

**Fig. 1:**
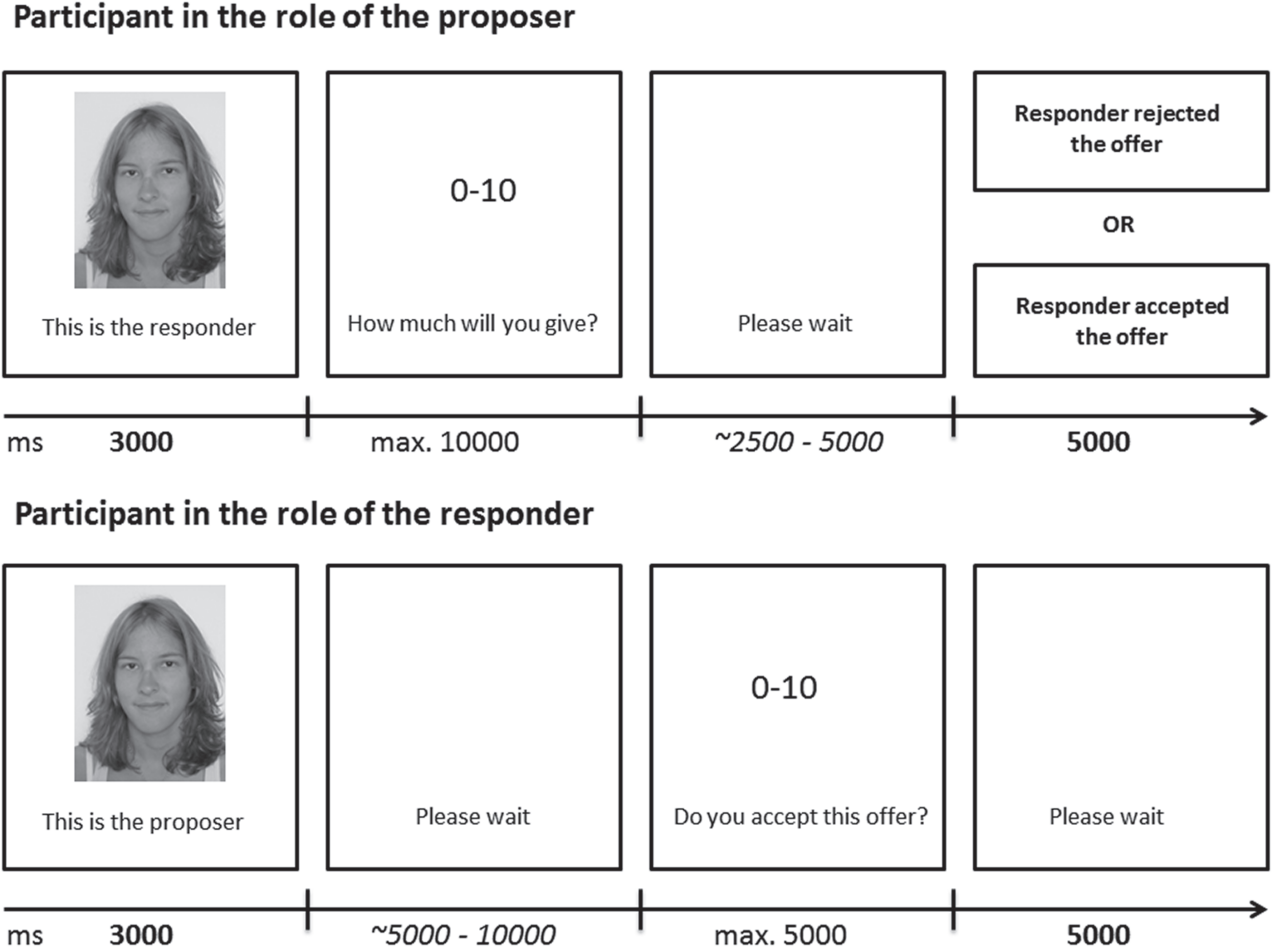
Depiction of the trial structure. Participants assumed the role of the proposer (upper) as well as in the role of responders (lower). Time scale is in milliseconds. Bold numbers represent fixed time windows. A tilde indicates a randomised time period and a ‘max’ indicates the maximum time participants had to react before the computer forced them to give the full amount of MU (proposer condition) or accept any amount from the other player (responder condition).

Participants were randomly assigned to the stimulation conditions. Twenty-three participants (12 female) received anodal stimulation, 23 (14 female) received cathodal stimulation and 21 (11 male) were in the sham condition. We collected demographic information via questionnaire and took a photo of each participant’s face. We told our participants that the other players would see the picture of them. They were told that they would play the UG via an online network with two other players (one at a time) from a different university and were presented with photos of the other players^1^ (Figure 1). This was in fact a cover story and the participants actually played against a predefined algorithm to ensure that every participant was confronted with all types of offers (fair [5:5] to unfair [9:1]) and with the same responses. In addition, it was emphasized that they would play with real money and would receive the amount of money earned during the UG after the experiment.

After participants read the instructions and rules of the UG on the computer screen, we started the tDCS stimulation. Participants were stimulated for 3 min before the trials started to familiarize them with the stimulation. During the first 10 trials, participants played in the role of proposer and were asked to split 10 monetary units (MUs; 1 MU = 0.10 euros) each trial between themselves and the responder. We presented the proposer condition first, in order to avoid a fairness bias because of the predefined offers in the responder condition. The participants were able to decide whether, and how, to share the money or keep all of it. Offers of less than three MUs were rejected. During the second 10 trials, participants played in the role of responder. As before, they played against the computer, this time receiving offers they could accept or reject. The artificial proposer followed a specific pseudorandomized and predefined ruleset: ‘fair’ offers of five and four MUs were contained to the first half; ‘unfair’ offers of two and one MUs were contained to the second half. This was intended to lead to progressively less fair offers over the course of time, in keeping with the standard pattern reported in previous research (Guth *et al.*, 1982).

 We designed the trial timing in a way that participants could expect a real opponent. In both conditions, participants saw a face, representing the other player, for 3000 ms. In the proposer condition, participants had 10 000 ms time to make an offer, while as a responder they had to wait a randomized period of time between 5000 and 10 000 ms. If proposers did not react within the 10 000 ms, they would automatically give the other player 10 MUs. Proposers were shown a waiting screen after that for a randomized period between 2500 and 5000 ms, while as responders they had 5000 ms to either accept or reject the offer. Finally, as the proposer participants saw the reaction of the other player for 5000 ms while in the role of the responder they had to wait for 5000 ms.

#### Stimulation parameters

TDCS causes a direct current to pass through the brain from an anode to a cathode fixed on the participant’s head. Anodal stimulation leads to enhanced cortical excitability, while cathodal stimulation decreases cortical activity (Nitsche *et al.*, 2008). With this ability to enhance and decrease brain activity, we used tDCS to investigate causal effects of human brain functioning on behaviour (Gandiga *et al.*, 2006). Compared with other methods of neurostimulation, tDCS has the advantage of more easily allowing placebo-controlled studies, because participants who receive tDCS have rarely been able to distinguish between real and sham stimulation (Gandiga *et al.*, 2006). We used a battery-driven direct current stimulator (neuroConn DC-Stimulator, Germany). For stimulation, we used two sponge electrodes soaked with an isotonic NaCl solution (0.9% NaCl). For stimulating the rTPJ, the active electrode (20 cm^2^) was placed on CP6, according to the international 10–20 system (Jasper, 1958), as it represents the area above the rTPJ (Herwig *et al.*, 2003; Donaldson *et al.*, 2015). The reference electrode (60 cm^2^) was placed over the left orbitofrontal cortex as it is a recommended and often used reference site (Woods *et al.*, 2016). Participants were stimulated by direct current with an intensity of 1 mA, resulting in a current density of 0.05 mA/cm^2^. The DC stimulation lasted 25 min. In the sham condition, electrodes were attached similarly to stimulation conditions, but tDCS lasted only 30 s.

### Results and discussion

**Fig. 2:**
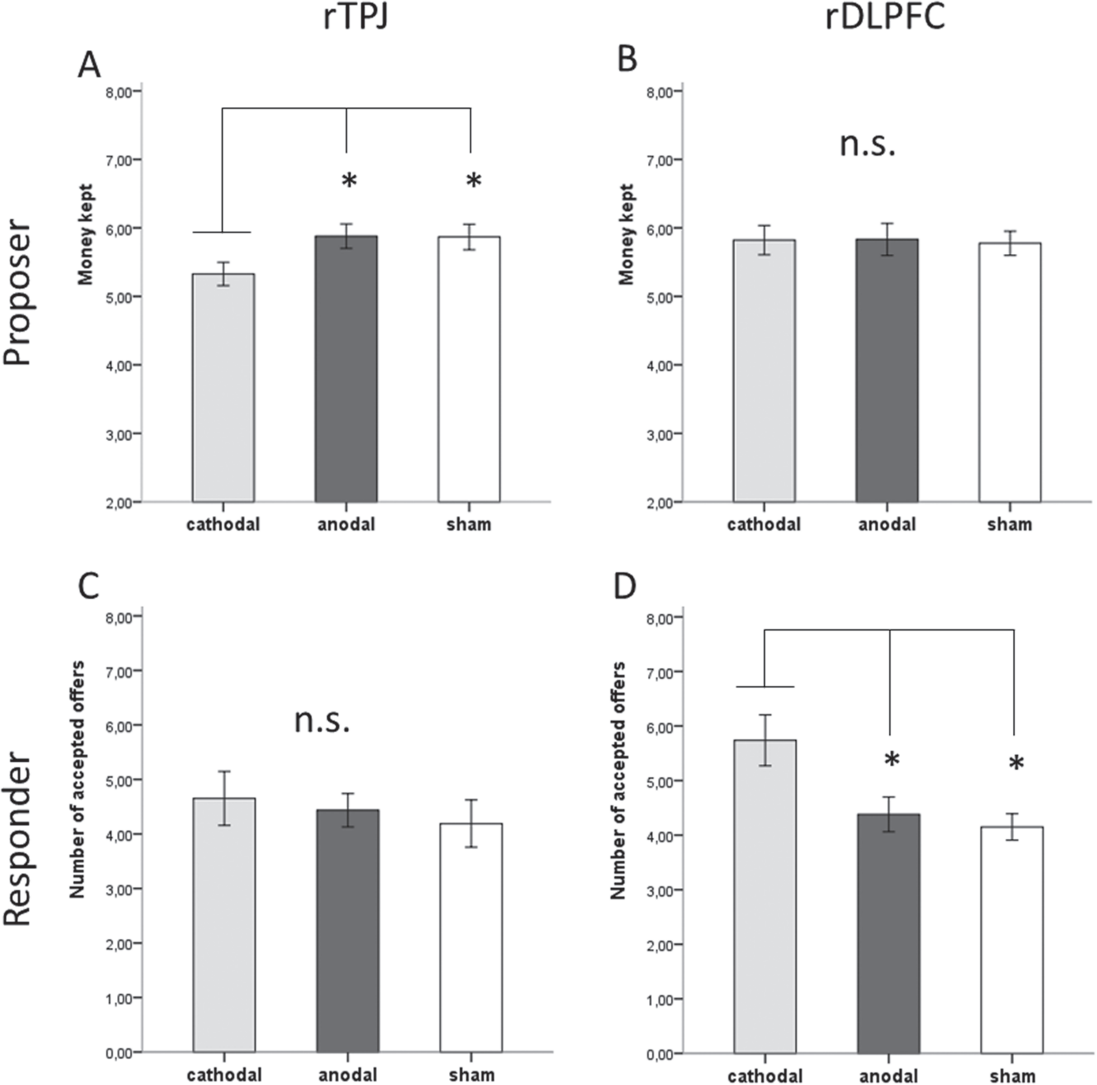
All error bars indicate ±1 Standard error (SE). Asterisks indicate *P* < 0.05. For proposers, (**A**) there was a significant difference between cathodal stimulation and anodal and sham stimulation in the effect on the average amount of money kept in the rTPJ stimulation groups. (**B**) There was no significant effect of stimulation condition on the average amount of money kept over 10 rounds in the rDLPFC stimulation groups. For responders, (**C**) there was no effect when rTPJ was stimulated. (**D**) There was a significant difference between cathodal stimulation and anodal and sham stimulation in the effect on the total number of offers accepted by responders.

We conducted an analysis of variance (ANOVA) with stimulation (anodal, cathodal, sham) as the between-subjects factor and the number of MUs kept by the proposer as a dependent variable. The ANOVA revealed a main effect of stimulation, *F*(2,64) = 3.19, *P* = 0.048, η^2^ = 0.09. A post hoc least significant difference (LSD) test showed that participants in the cathodal condition kept less money (*M* = 5.32, s.d. = 0.82) compared to participants in the anodal condition (*M* = 5.88, s.d. = 0.86, *P* < 0.03) and the sham condition (*M* = 5.87, s.d. = 0.85, *P* < 0.04; Figure 2A). The anodal condition did not differ significantly from the sham condition (*P* < 0.98). We then analysed the total number of rejections as a dependent variable in another ANOVA. The between-subjects factor was again stimulation (anodal, cathodal and sham). There was no main effect of stimulation on the rejection rates in the responder condition, *F*(2,67) = 0.29, *P* = 0.74, η^2^ = 0.009 (Figure 2C).

The cathodal stimulation of the rTPJ led to a significant decrease in the amount of money kept by the proposers. So, in line with our hypothesis, the inhibition of rTPJ led to offers closer to an equal distribution. The reduction of rTPJ activity may have made it more difficult for the participant to differentiate between their own and the other player’s material payoff.

In the responder condition, there was no indication that the rTPJ was actively involved in the conflict between selfish impulses and fairness norms. This lack of a clear effect brings up the question of the necessity of taking the perspective as a responder. It may not require perspective taking, since participants have to react only to the offer itself and not necessarily to another person. In the proposer condition, in contrast, taking the other perspective is necessary for the outcome of the proposer’s own decision; that is, the other player’s perspective is relative to the proposer’s own perspective. In Experiment 2, we compared these results to the regulatory functions of the rDLPFC.

## Experiment 2: rDLPFC

### Methods

#### Participants

Sixty-seven healthy participants (34 females), mean age 21.65 years (s.d. = 2.9), gave written informed consent and were instructed about the general procedure and potential side effects of tDCS. The study was approved by the ethics committee of the University of Salzburg. Additionally, we explained that the participants’ data would be treated anonymously and that participants could stop their participation in the experiment whenever they wanted without giving an explanation. As a reward for taking part in this experiment, participants received the money earned in the UG. Three participants had to be excluded because of technical problems with the recording of the responses.

#### Procedure

Participants were randomly assigned to the stimulation conditions. Twenty-three participants (12 male) received cathodal stimulation, 21 (13 female) received anodal stimulation and 20 (10 female) were in the sham condition. The procedure was the same as in Experiment 1 except that after the UG but before the end of the tDCS stimulation, participants were presented with every possible offer and asked to report on a 7-point scale to what extent they perceived the presented offer as fair or unfair (1 = *very unfair*, 7 = *very fair*).

#### Stimulation parameters

To stimulate the rDLPFC, the active electrode (35 cm^2^) was placed on F4, while the reference electrode (60 cm^2^) was placed over the left orbitofrontal cortex. Participants were stimulated by direct current with an intensity of 1.8 mA, which translates into a current density of 0.051 mA/cm^2^. All other parameters and specifications were parallel to Experiment 1.

### Results and discussion

To test if the tDCS stimulation of the rDLPFC affected the size of proposals, we used an ANOVA with the between-subjects factor stimulation (cathodal, anodal and sham). The dependent variable was how much money the participants kept for themselves. We found no significant main effect of stimulation, *F*(2,61) = 0.19, *P* = 0.981, η^2^ = 0.001 (Figure 2B).

We then analysed the total number of rejections as a dependent variable in a one-way ANOVA with the between-subjects factor stimulation (anodal, cathodal and sham). We found a significant main effect of stimulation, *F*(2,61) = 5.62, *P* < 0.01, η^2^ = 0.16. A post hoc LSD test revealed that participants in the cathodal stimulation condition (*M* = 5.74, s.d. = 2.24) accepted more offers than participants in the anodal (*M* = 4.38, s.d. = 1.47, *P* < 0.01) and sham (*M* = 4.15, s.d. = 1.09, *P* < 0.003) conditions (Figure 2D). The anodal condition did not differ from the sham condition (*P* = 0.67). Parallel to the results of Knoch *et al*. (2008), we also found no effect on the fairness judgement using a multivariate ANOVA with stimulation (anodal, cathodal and sham) as independent and the fairness judgements as dependent variables (*P* = 0.44 to 0.86).

As previously shown, participants’ propensity to punish unfair offers was reduced by cathodal stimulation. This is true even when using computer-generated offers and a different trial structure from former studies (Knoch *et al.*, 2008).^2^ Despite the successful stimulation effect in the responder condition, we did not find any effect on the offer size in the proposer condition. This means, stimulation of the rDLPFC had no effect on the amount of money participants gave away. This questions the idea that the rDLPFC inhibits selfish impulses in general if one assumes that selfish impulses should play a role in the behaviour of proposers. This is further supported by the fact that the perceived fairness of the offers is not influenced by the stimulation. It is not clear which functional aspect of the rDLPFC causes the rejection of unfair offers. One possible explanation could be that only the responder was confronted with a stimulus (the unfair offer) that causes a negative affective state (Sanfey *et al.*, 2003; Civai *et al.*, 2010) and thus a conflict between this state and the self-interest of the participant. Cathodal stimulation would disrupt this conflict. So, it could be that the prepotent function of the rDLPFC is not to suppress selfish impulses in favour of fairness norms but in favour of an anger reaction to a negative treatment, which was absent in the role of the proposer.

## General discussion

Results show a clear difference between the functions of the rTPJ and the rDLPFC in proposer and responder trials. The stimulation of the rTPJ led to fairer offers in the proposer condition; it had no effect in the responder condition. For the rDLPFC, it was the other way around, displaying a clear double dissociation between the functions of those two areas. As a proposer in the UG, being fair minimizes the risk of being rejected and simultaneously maximizes one’s own outcome. It is possible and, given the typically fair shares, likely that fairness norms are integrated into this decision and the perspective of the other player is considered.

First, we look at the rTPJ cathodal stimulation effect in the proposer condition, which was the result of either the distinction between the participants’ own perspective and that of the other player or the simulation of the other’s perspective being disrupted. Another explanation would be that because people tend to try to avoid risks, especially in uncertain situations (Kühberger, 1998), the inhibition of the rTPJ might have led to a less clear concept of the others’ actions and thus caused more risk-averse behaviour. We cannot rule out the possibility that alternative non-social functions of the rTPJ like attention (Geng and Vossel, 2013) or mathematical cognition (Butterworth and Walsh, 2011) are the cause of the effect. Since the UG is inherently also a simple arithmetic task, it is possible that inhibition of the rTPJ led to a simple 5/5 heuristic because other splits might be more challenging. While this must be kept in mind, the vast majority of recent studies using economic games focus on the social cognitive side of the rTPJ (Baumgartner *et al.*, 2012; Morishima *et al.*, 2012; Baumgartner *et al.*, 2014; Guo *et al.*, 2014).

The rTPJ seems not to be involved in the conflict between self-interest and fairness or any form of social punishment, given the lack of an effect in the responder condition, where this conflict should be prominent. The absence of an effect for responders leaves the question if participants need to process the other player’s perspective when reacting to an offer. This is in contrast with imaging studies, showing rTPJ activity in the responder condition in some variations of the UG (Guroglu *et al.*, 2010). However, in the studies we reviewed, we found the rTPJ was only involved when participants were, for example, forced to reject an unfair offer even if the other proposer had, by design of the experiment, no choice than to give an unfair amount (Guroglu *et al.*, 2010; Guroglu *et al.*, 2011) or if multiple responders had to compete with one another (Halko *et al.*, 2009). In these situations, additional considerations of other perspectives or even additional perspectives must be made by the participants. Our results indicate that, at least in a standard UG with multiple rounds, the functions of the rTPJ seem not to be of significant relevance for participants in the role of a responder.

Considering the rDLPFC in this light, we see, in fact, that it was involved in processing the conflict of fairness norms *vs* selfish impulses, at least in the responder condition as described in previous studies (van’t Wout *et al.*, 2005; Knoch *et al.*, 2008). Surprisingly, this study shows that there is no such conflict for proposers. If one assumes that this conflict must exist for proposers, then this would contradict the claim that the rDLPFC processes the integration of fairness norms.

### Limitations

One can argue that the responder condition in Experiment 1 was influenced by the behaviour in the previously presented proposer condition. This might have created an expectation for the responders (Chang and Sanfey, 2009). Nevertheless, the results showed the same behavioural pattern as found by Knoch *et al.* (2008). However, the criticism is valid and might have caused the participants in their role as responder to be less spontaneous. To address this, we also tested if the number of rejections in the responder condition was in any way correlated with the amount of money shared in the proposer condition. We could not find any significant correlations that would support that argument.

Also, unlike earlier studies on bargaining behaviour there was no condition in which the participants thought to play against a computer to control for the human element (Sanfey *et al.*, 2003; Knoch *et al.*, 2006). This could limit our interpretation, especially of the rTPJ results and our assumption that we decreased the ability of self–other distinction. Future studies should include non-human control conditions for clarifying the role of the rTPJ, especially with regard to its involvement in arithmetic.

A similar problem is caused by the limited focality of tDCS: When large electrodes are used, tDCS might stimulate not only the intended brain regions but also adjacent ones (Nitsche *et al.*, 2008). This is especially true for smaller areas such as the rTPJ. We addressed this by using smaller electrodes than is common, but areas adjacent to the rTPJ, that might have different functions for economic decision making, may have been stimulated (for an overview see, Hetu *et al.*, 2012). Future studies should address this problem by using a more focal stimulation method such as high definition tDCS (Villamar *et al.*, 2013).

There was also no difference between the anodal and sham conditions in either experiment, parallel to other recent studies investigating the rTPJ in bargaining (Blair-West *et al.*, 2018). It is possible that in a task such as the UG where the capabilities of an area are needed and therefore used to capacity, there is no room for improvement. The anodal stimulation may not have had any effect on an already fully activated area.

## Conclusion

Our results indicate that the two target areas of this study, rTPJ and rDLPFC, process different aspects of bargaining. The rTPJ processes the distinction between the perspectives of both players when they are necessary, and they seem to be necessary primarily in the proposer condition. We furthermore replicated that the rDLPFC has a role as a self-regulatory system reacting to unfair offers. However, this cannot be extended to the proposer condition and thus not to the integration of fairness norms or overriding self-interest per se. This strengthens the notion that the rDLPFC is most likely responsible for self-control when faced with unfairness. Finally, the observed double dissociation between rTPJ and rDLPFC using tDCS suggests that the function of one area may be independent from the other.

## Footnotes

1 The photos of the other players were taken in a pilot study, where they were rated and matched for attractiveness. We used one photo of a male and one of a female.

2 All included participants in the present study stated after the experiment that they believed that they were playing with a real human opponent.

## References

[ref1] Artinger F., Exadaktylos F., Koppel H., Saaksvuori L. (2014). In others’ shoes: do individual differences in empathy and theory of mind shape social preferences. *PLoS ONE*, 9(4), e92844. 24743312 10.1371/journal.pone.0092844 PMC3990498

[ref2] Baumgartner T., Gotte L., Gugler R., Fehr E. (2012). The mentalizing network orchestrates the impact of parochial altruism on social norm enforcement. *Human Brain Mapping*, 33(6), 1452–69. 21574212 10.1002/hbm.21298 PMC6870290

[ref3] Baumgartner T., Schiller B., Rieskamp J., Gianotti L.R., Knoch D. (2014). Diminishing parochialism in intergroup conflict by disrupting the right temporo-parietal junction. *Social Cognitive and Affective Neuroscience*, 9(5), 653–60. 23482623 10.1093/scan/nst023 PMC4014096

[ref4] Been G., Ngo T.T., Miller S.M., Fitzgerald P.B. (2007). The use of tDCS and CVS as methods of non-invasive brain stimulation. *Brain Research Reviews*, 56(2), 346–61. 17900703 10.1016/j.brainresrev.2007.08.001

[ref5] Blair-West L.F., Hoy K.E., Hall P.J., Fitzgerald P.B., Fitzgibbon B.M. (2018). No change in social decision-making following transcranial direct current stimulation of the right temporoparietal junction. *Frontiers in Neuroscience*, 12, 258. 29725288 10.3389/fnins.2018.00258 PMC5917038

[ref6] Butterworth B., Walsh V. (2011). Neural basis of mathematical cognition. *Current Biology*, 21(16), R618–21. 21854998 10.1016/j.cub.2011.07.005

[ref7] Camerer C., Thaler R.H. (1995). Anomalies: ultimatums, dictators and manners. *Journal of Economic Perspectives*, 9(2), 209–19.

[ref8] Chang L.J., Sanfey A.G. (2009). Unforgettable ultimatums? Expectation violations promote enhanced social memory following economic bargaining. *Frontiers in Behavioral Neuroscience*, 3, 36. 19876405 10.3389/neuro.08.036.2009 PMC2769546

[ref9] Civai C., Corradi-Dell'Acqua C., Gamer M., Rumiati R.I. (2010). Are irrational reactions to unfairness truly emotionally-driven? Dissociated behavioural and emotional responses in the ultimatum game task. *Cognition*, 114(1), 89–95. 19786275 10.1016/j.cognition.2009.09.001

[ref10] Civai C., Miniussi C., Rumiati R.I. (2015). Medial prefrontal cortex reacts to unfairness if this damages the self: a tDCS study. *Social Cognitive and Affective Neuroscience*, 10(8), 1054–1060. 25552567 10.1093/scan/nsu154 PMC4526475

[ref11] Decety J., Lamm C. (2007). The role of the right temporoparietal junction in social interaction: how low-level computational processes contribute to meta-cognition. *Neuroscientist*, 13(6), 580–93. 17911216 10.1177/1073858407304654

[ref12] Decety J., Sommerville J.A. (2003). Shared representations between self and other: a social cognitive neuroscience view. *Trends in Cognitive Sciences*, 7(12), 527–533. 14643368 10.1016/j.tics.2003.10.004

[ref13] Donaldson P., Rinehart N.J., Enticott P.G. (2015). Noninvasive stimulation of the temporoparietal junction: a systematic review. *Neuroscience and Biobehavioral Reviews*, 55, 547–72. doi: 10.1016/j.neubiorev.2015.05.017. 26073069

[ref14] Duncan J., Owen A.M. (2000). Common regions of the human frontal lobe recruited by diverse cognitive demands. *Trends in Neurosciences*, 23(10), 475–483. 11006464 10.1016/s0166-2236(00)01633-7

[ref15] Fehr E., Camerer C.F. (2007). Social neuroeconomics: the neural circuitry of social preferences. Trends in Cognitive Sciences, 11(10), 419–427. 17913566 10.1016/j.tics.2007.09.002

[ref16] Gandiga P.C., Hummel F.C., Cohen L.G. (2006). Transcranial DC stimulation (tDCS): a tool for double-blind sham-controlled clinical studies in brain stimulation. *Clinical Neurophysiology*, 117, 845–50. 16427357 10.1016/j.clinph.2005.12.003

[ref17] Geng J.J., Vossel S. (2013). Re-evaluating the role of TPJ in attentional control: contextual updating. *Neuroscience & Biobehavioral Reviews*, 37(10 Pt 2), 2608–20. 23999082 10.1016/j.neubiorev.2013.08.010 PMC3878596

[ref18] Grecucci A., Giorgetta C., van't Wout M., Bonini N., Sanfey A.G. (2013). Reappraising the ultimatum: an fMRI study of emotion regulation and decision making. *Cerebral Cortex*, 23(2), 399–410. 22368088 10.1093/cercor/bhs028

[ref19] Guo X., Zheng L., Cheng X., et al. (2014). Neural responses to unfairness and fairness depend on self-contribution to the income. *Social Cognitive and Affective Neuroscience*, 9(10), 1498–505. 23946001 10.1093/scan/nst131 PMC4187258

[ref20] Guroglu B., van den Bos W., Rombouts S.A., Crone E.A. (2010) Unfair? It depends: neural correlates of fairness in social context. *Social Cognitive and Affective Neuroscience*, 5(4), 414–423. 20350933 10.1093/scan/nsq013 PMC2999761

[ref21] Guroglu B., van den Bos W., van Dijk E., Rombouts S.A.R.B., Crone E.A. (2011) Dissociable brain networks involved in development of fairness considerations: understanding intentionality behind unfairness. *NeuroImage*, 57(2), 634–641. 21554961 10.1016/j.neuroimage.2011.04.032

[ref22] Guth W., Schmittberger R., Schwarze B. (1982). An experimental analysis of ultimatum bargaining. *Journal of Economic Behavior & Organization*, 3(4), 367–388.

[ref23] Hadland K.A., Rushworth M.F., Passingham R.E., Jahanshahi M., Rothwell J.C. (2001). Interference with performance of a response selection task that has no working memory component: an rTMS comparison of the dorsolateral prefrontal and medial frontal cortex. *Journal of Cognitive Neuroscience*, 13(8), 1097–1108. 11784448 10.1162/089892901753294392

[ref24] Halko M.-L., Hlushchuk Y., Hari R., Schuermann M. (2009). Competing with peers: mentalizing-related brain activity reflects what is at stake. *NeuroImage*, 46(2), 542–548. 19385019 10.1016/j.neuroimage.2009.01.063

[ref25] Hare T.A., Camerer C.F., Rangel A. (2009). Self-control in decision-making involves modulation of the vmPFC valuation system. *Science*, 324(5927), 646–648. 19407204 10.1126/science.1168450

[ref26] Herwig U., Satrapi P., Schonfeldt-Lecuona C. (2003). Using the international 10-20 EEG system for positioning of transcranial magnetic stimulation. *Brain Topography*, 16(2), 95–99. 14977202 10.1023/b:brat.0000006333.93597.9d

[ref27] Hetu S., Taschereau-Dumouchel V., Jackson P.L. (2012). Stimulating the brain to study social interactions and empathy. *Brain Stimulation*, 5(2), 95–102. 22503509 10.1016/j.brs.2012.03.005

[ref28] Jasper H.H. (1958). Report of the committee on methods of clinical examination in electroencephalography. *Electroencephalography and Clinical Neurophysiology*, 10(2), 370–375.

[ref29] Kanngiesser P., Warneken F. (2012). Young children consider merit when sharing resources with others. *PLoS ONE*, 7(8), e43979. 22952834 10.1371/journal.pone.0043979 PMC3430625

[ref30] Kirman A., Teschl M. (2010). Selfish or selfless? The role of empathy in economics. *Philosophical Transactions of the Royal Society of London. Series B, Biological Sciences*, 365(1538), 303–317. 20026468 10.1098/rstb.2009.0192 PMC2827457

[ref31] Knoch D., Nitsche M.A., Fischbacher U., Eisenegger C., Pascual-Leone A., Fehr E. (2008). Studying the neurobiology of social interaction with transcranial direct current stimulation—the example of punishing unfairness. *Cerebral Cortex*, 18(9), 1987–1990. 18158325 10.1093/cercor/bhm237

[ref32] Knoch D., Pascual-Leone A., Meyer K., Treyer V., Fehr E. (2006). Diminishing reciprocal fairness by disrupting the right prefrontal cortex. *Science*, 314(5800), 829–32. 17023614 10.1126/science.1129156

[ref33] Kühberger A. (1998). The influence of framing on risky decisions: a meta-analysis. *Organizational Behavior and Human Decision Processes*, 75(1), 23–55. 9719656 10.1006/obhd.1998.2781

[ref34] Leventhal G.S., Popp A.L., Sawyer L. (1973). Equity or equality in children’s allocation of reward to other persons. *Child Development*, 44(4), 753.

[ref35] Morelli S.A., Lieberman M.D. (2013). The role of automaticity and attention in neural processes underlying empathy for happiness, sadness, and anxiety. *Frontiers in Human Neuroscience*, 7, 160. doi: 10.3389/fnhum.2013.00160. 23658538 PMC3647144

[ref36] Morewedge C.K., Krishnamurti T., Ariely D. (2014). Focused on fairness: alcohol intoxication increases the costly rejection of inequitable rewards. *Journal of Experimental Social Psychology*, 50, 15–20.

[ref37] Morishima Y., Schunk D., Bruhin A., Ruff C.C., Fehr E. (2012). Linking brain structure and activation in temporoparietal junction to explain the neurobiology of human altruism. *Neuron*, 75(1), 73–79. 22794262 10.1016/j.neuron.2012.05.021

[ref38] Nitsche M.A., Cohen L.G., Wassermann E.M., et al. (2008). Transcranial direct current stimulation: state of the art 2008. *Brain Stimulation*, 1(3), 206–223. 20633386 10.1016/j.brs.2008.06.004

[ref39] Perner J., Aichhorn M., Kronbichler M., Staffen W., Ladurner G. (2006). Thinking of mental and other representations: the roles of left and right temporo-parietal junction. *Social Neuroscience*, 1(3–4), 245–258. 18633791 10.1080/17470910600989896

[ref40] Rilling J.K., Sanfey A.G., Aronson J.A., Nystrom L.E., Cohen J.D. (2004). The neural correlates of theory of mind within interpersonal interactions. *NeuroImage*, 22(4), 1694–1703. 15275925 10.1016/j.neuroimage.2004.04.015

[ref41] Sanfey A.G. (2007). Social decision-making: insights from game theory and neuroscience. *Science*, 318(5850), 598–602. 17962552 10.1126/science.1142996

[ref42] Sanfey A.G., Rilling J.K., Aronson J.A., Nystrom L.E., Cohen J.D. (2003). The neural basis of economic decision-making in the ultimatum game. *Science*, 300(5626), 1755–1758. 12805551 10.1126/science.1082976

[ref43] Schulte-Ruther M., Markowitsch H.J., Shah N.J., Fink G.R., Piefke M. (2008). Gender differences in brain networks supporting empathy. *NeuroImage*, 42(1), 393–403. 18514546 10.1016/j.neuroimage.2008.04.180

[ref44] Sellaro R., Nitsche M.A., Colzato L.S. (2016) The stimulated social brain: effects of transcranial direct current stimulation on social cognition. In: Kingstone A., Miller M.B., editors. *Year in Cognitive Neuroscience*, Oxford: Blackwell Science Publishing. 10.1111/nyas.13098 27206250

[ref45] Singer T. (2006). The neuronal basis and ontogeny of empathy and mind reading: review of literature and implications for future research. Neuroscience and Biobehavioral Reviews, 30(6), 855–863. 16904182 10.1016/j.neubiorev.2006.06.011

[ref46] Stallen M., Sanfey A.G. (2013). The cooperative brain. *Neuroscientist*, 19(3), 292–303. 23300215 10.1177/1073858412469728

[ref47] Tabibnia G., Satpute A.B., Lieberman M.D. (2008). The sunny side of fairness: preference for fairness activates reward circuitry (and disregarding unfairness activates self-control circuitry). *Psychological Science*, 19(4), 339–347. 18399886 10.1111/j.1467-9280.2008.02091.x

[ref48] Thair H., Holloway A.L., Newport R., Smith A.D. (2017). Transcranial direct current stimulation (tDCS): a beginner’s guide for design and implementation. *Frontiers in Neuroscience*, 11, 641. 29213226 10.3389/fnins.2017.00641 PMC5702643

[ref49] van't Wout M., Kahn R.S., Sanfey A.G., Aleman A. (2005). Repetitive transcranial magnetic stimulation over the right dorsolateral prefrontal cortex affects strategic decision-making. *Neuroreport*, 16(16), 1849–1852. 16237340 10.1097/01.wnr.0000183907.08149.14

[ref50] van’t Wout M., Kahn R.S., Sanfey A.G., Aleman A. (2006). Affective state and decision-making in the ultimatum game. *Experimental Brain Research*, 169(4), 564–568. 16489438 10.1007/s00221-006-0346-5

[ref51] Van Overwalle F. (2009). Social cognition and the brain: a meta-analysis. *Human Brain Mapping*, 30(3), 829–858. 18381770 10.1002/hbm.20547 PMC6870808

[ref52] Villamar M.F., Volz M.S., Bikson M., Datta A., Dasilva A.F., Fregni F. (2013). Technique and considerations in the use of 4×1 ring high-definition transcranial direct current stimulation (HD-tDCS). *Journal of Visualized Experiments*, 77, e50309. doi: 10.3791/50309. PMC3735368 23893039

[ref53] Woods A.J., Antal A., Bikson M., et al. (2016). A technical guide to tDCS, and related non-invasive brain stimulation tools. Clinical Neurophysiology, 127(2), 1031–1048. 26652115 10.1016/j.clinph.2015.11.012 PMC4747791

